# Supplementary material for: Cross-talk between engineered Clostridium acetobutylicum and Clostridium ljungdahlii in syntrophic cocultures enhances isopropanol and butanol production
Source: Front Microbiol. 2025 Oct 6;16:1674318. doi: 10.3389/fmicb.2025.1674318 (PMC12536658; doi:10.3389/fmicb.2025.1674318)
Supplement: Supplementary file 1 [file Data_Sheet_1.pdf]

# *Supplementary Material*

## 1 Table S1

**Calculated acetone/IPA yields with all sugars consumed (glucose and fructose) or with glucose only for each experimental condition.** The theoretical monoculture (MC)-only yield is 0.6, so the presence of *Clj* in cocultures (CC) leads to that value being exceeded in serum bottles or to the monoculture yields being nearly doubled in bioreactors (BRs, 0.24-0.25 to 0.50-0.53 in high-SCD conditions).

| Letter | Yields considering glucose and fructose | Yields considering glucose |
|---|---|---|
| Bottled MC – $N_2$ | 0.42 | 0.43 |
| Bottled CC – $N_2$ | 0.56 | 0.61 |
| Bottled CC – $H_2$ | 0.53 | 0.59 |
| Bottled CC – $H_2/CO_2$ | 0.68 | 0.76 |
| Low-SCD MC BRs | 0.29 | 0.31 |
| High-SCD MC BRs | 0.24 | 0.25 |
| Low-SCD CC BRs | 0.46 | 0.48 |
| High-SCD CC BRs | 0.50 | 0.53 |
