# Supplementary material for: Cross-talk between engineered Clostridium acetobutylicum and Clostridium ljungdahlii in syntrophic cocultures enhances isopropanol and butanol production
Source: Front Microbiol. 2025 Oct 6;16:1674318. doi: 10.3389/fmicb.2025.1674318 (PMC12536658; doi:10.3389/fmicb.2025.1674318)
Supplement: Supplementary file 2 [file Data_Sheet_2.pdf]

Supplementary Material

1 Appendix 1: Stoichiometric Coefficient Calculations and Details

<u>Chemical Species in CC Model</u>

Extracellular metabolites: (accumulation determined by HPLC)

1. glucose
2. fructose
3. acetate
4. ethanol
5. isopropanol
6. butyrate
7. acetone
8. butanol

C. acetobutylicum **intracellular metabolites: (assumed to not accumulate)**

9. pyruvate-(Cac)
10. acetyl-CoA-(Cac)
11. acetoacetyl-CoA-(Cac)
12. butyryl-CoA-(Cac)
13. NADH-(Cac)
14. Fd_{red}-(Cac)

C. ljungdahlii **intracellular metabolites: (assumed to not accumulate)**

15. pyruvate-(Clj)
16. acetyl-CoA-(Clj)
17. EC-(Clj)

<u>Reactions in the CC Model</u>

Reaction 1: rGLY1.Cac

glucose + 2 NAD^+-(Cac) → 2 pyruvate-(Cac) + 2 NADH-(Cac)

Reaction 2: rGLY2.Cac

pyruvate-(Cac) → acetyl-CoA-(Cac) + Fd_{red}-(Cac) + CO_2

Reaction 3: rFDNH

Fd_{red}-(Cac) + NAD^+-(Cac) → Fd_{ox}-(Cac) + NADH-(Cac)

Reaction 4: rHYD.Cac

Fd_{red}-(Cac) + 2 H^+ → Fd_{ox}-(Cac) + H_2

Reaction 5: rPTAAK.Cac

acetyl-CoA-(Cac) → acetate

Reaction 6: rETOH

acetyl-CoA-(Cac) + 2 NADH-(Cac) → ethanol + 2 NAD^+-(Cac)

Reaction 7: rTHL

2 acetyl-CoA-(Cac) → acetoacetyl-CoA-(Cac)

Reaction 8: rBYCA

acetoacetyl-CoA-(Cac) + Fd_{ox}-(Cac) + 3 NADH-(Cac) → butyryl-CoA-(Cac) + Fd_{red}-(Cac) + 3 NAD^+-(Cac)

Reaction 9: rPTBBK

butyryl-CoA-(Cac) → butyrate

Reaction 10: rBUOH

butyryl-CoA-(Cac) + 2 NADH-(Cac) → butanol + 2 NAD^+-(Cac)

Reaction 11: rBYUP

butyrate + acetoacetyl-CoA-(Cac) → acetone + butyryl-CoA-(Cac) + CO_2

Reaction 12: rACUP

acetate + acetoacetyl-CoA-(Cac) → acetone + acetyl-CoA-(Cac) + CO_2

Reaction 13: rGLY1.Clj

fructose + 2 EC_{ox}-(Clj) → 2 pyruvate-(Clj) + 2 EC_{red}-(Clj)

Reaction 14: rGLY2.Clj

pyruvate-(Clj) + EC_{ox}-(Clj) → acetyl-CoA-(Clj) + EC_{red}-(Clj) + CO_2

Reaction 15: rPTAAK.Clj

acetyl-CoA-(Clj) → acetate

Reaction 16: rHYD.Clj

H_2 + EC_{ox}-(Clj) → 2 H^+ + EC_{red}-(Clj)

Reaction 17: rWLP

$2\ CO_2 + 4\ EC_{red}$-(Clj) → acetyl-CoA-(Clj) + $4\ EC_{ox}$-(Clj)

Reaction 18: rSADH

acetone + EC_{red}-(Clj) → isopropanol + EC_{ox}-(Clj)

Details

The specific intracellular Electron Carrier (EC_{ox}/EC_{red}) in *Clj* is unimportant because the 'rnf/ATPase' complex allows interconversion between NADH and Fd_{red} at the expense of ATP, which is not accounted for. This is in contrast to *Cac*'s electron carriers which are specified as being NAD^+/NADH or Fd_{ox}/Fd_{red}.

CO_2 and H_2 are included in the above equations for convenience, but their accumulation cannot be used to constrain the model's solution because their accumulation was not measured.

In theory, this is an under-defined matrix, because there are 18 unique reactions, and only 17 metabolites. Therefor it could generally find a 'perfect' solution, where the sum of the square residuals (SSR) is 0. However, that may involve running certain reactions in reverse, which may or may not be physiologically possible. In practice, I use the non-negative least squares (nnls) solver in SciPy, such that no element of the x-matrix can be less than 0. This means that the solution space may not always contain a particular solution in which the SSR is 0. When this occurs, the solver relies on a minimum finding algorithm to find the particular solution with the smallest possible SSR.
