# Supplementary material for: Cross-talk between engineered Clostridium acetobutylicum and Clostridium ljungdahlii in syntrophic cocultures enhances isopropanol and butanol production
Source: Front Microbiol. 2025 Oct 6;16:1674318. doi: 10.3389/fmicb.2025.1674318 (PMC12536658; doi:10.3389/fmicb.2025.1674318)
Supplement: Supplementary file 3 [file Data_Sheet_3.pdf]

Supplementary Material

1 Figure S1

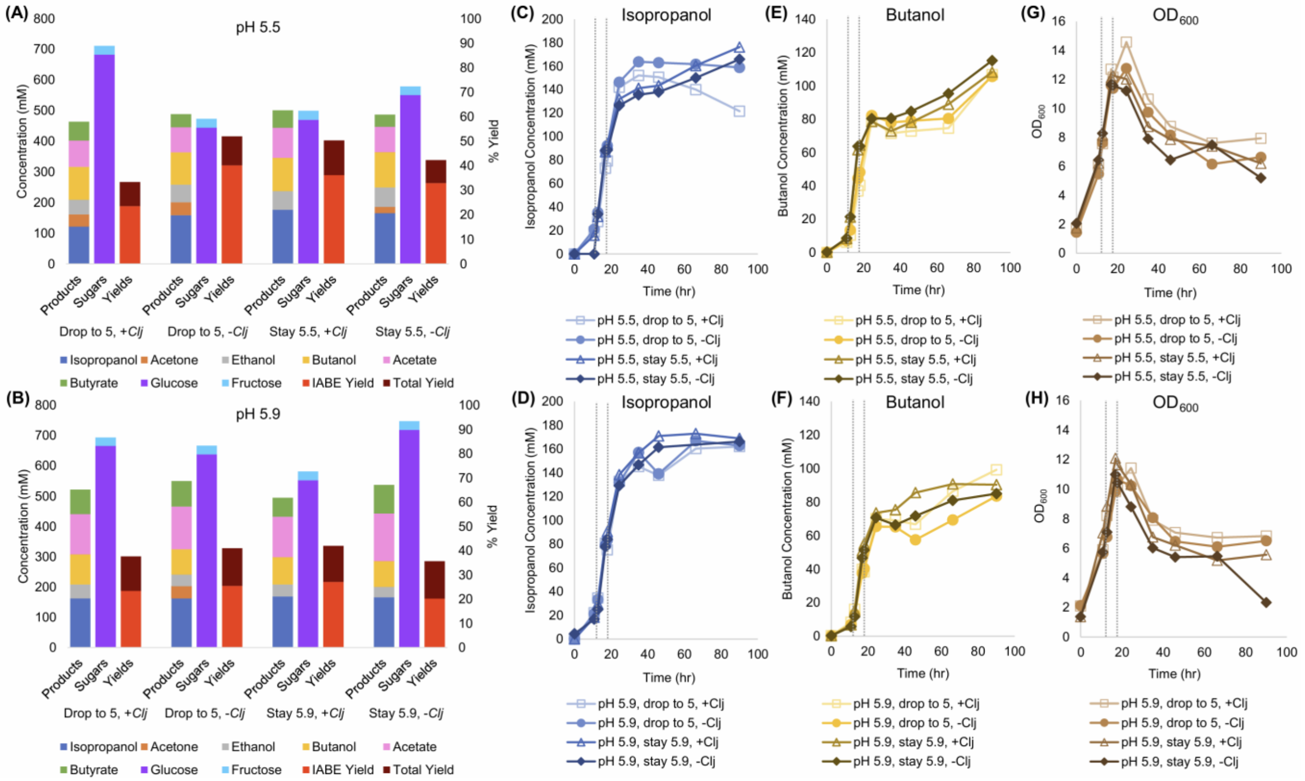

Figure S1: Metabolite production and yields, and sugar consumption to analyze pH setpoints and the impact of supplemental *Clj* cells for *Cac* and *Clj* cocultures in bioreactors. The vertical, short-dashed lines represent glucose additions to the bioreactors at 12 and 19 hours. (A-B) Summary of product concentrations, sugar consumption, and yields at a set pH of 5.5 (A) and at a set pH of 5.9 (B). IABE yield refers only to IPA, BuOH, EtOH, and acetone. The total yield also includes butyrate and acetate. (C-D) The IPA formation kinetics of the pH 5.5 (C) and pH 5.9 (D) cocultures with ("drop to 5") and without ("stay at") a pH setpoint drop to 5.0 and additional *Clj* (+*Clj*; no additional *Clj* is -*Clj*). (E-F) The BuOH formation kinetics of the pH 5.5 (E) and pH 5.9 (F) cocultures. (G-H) The total biomass (OD_{600}) of the pH 5.5 (G) and pH 5.9 (H) cocultures.

2 Figure S2

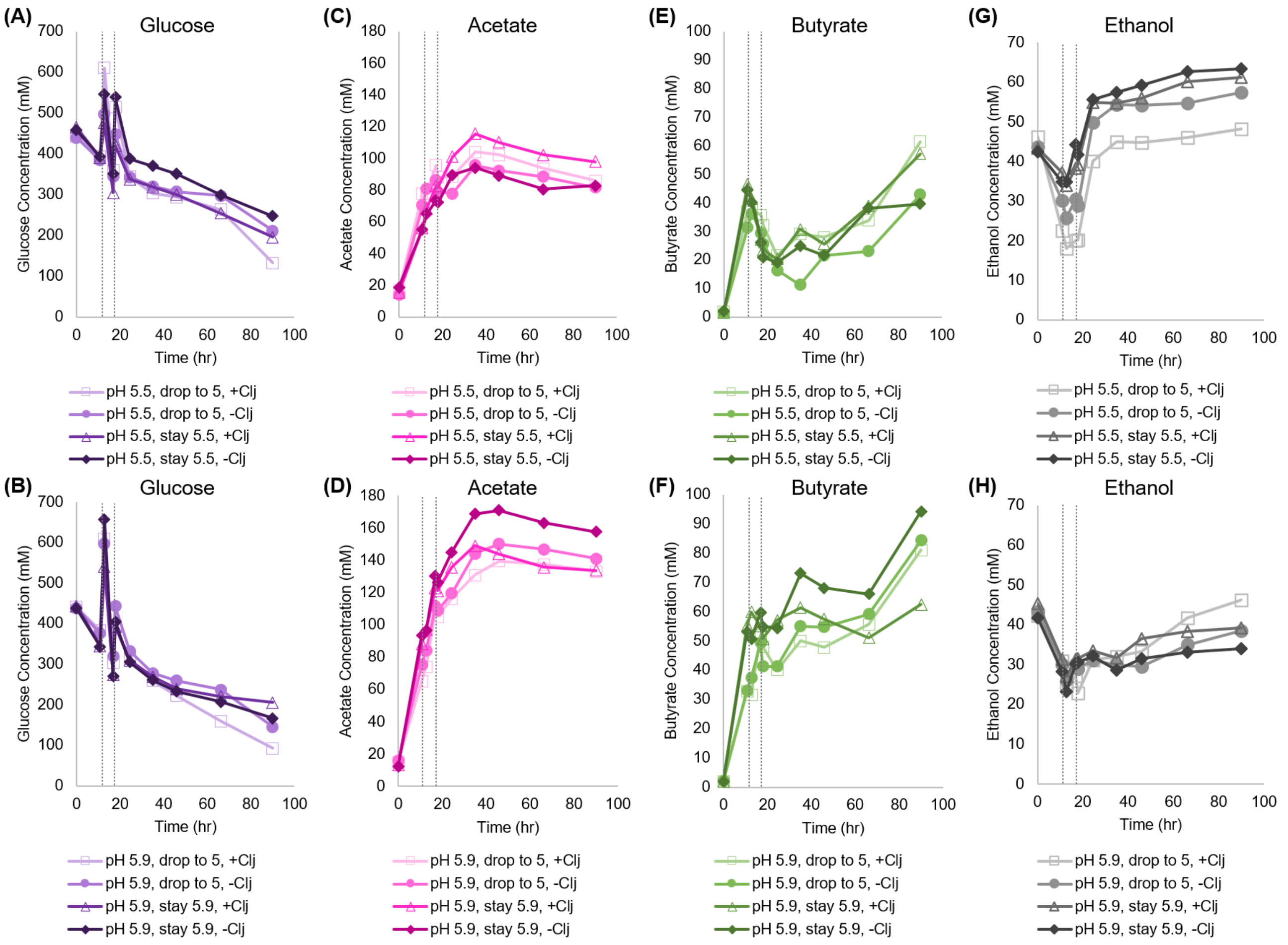

Figure S2: Kinetics of glucose, acetate, butyrate, and ethanol in fermentations to analyze pH setpoints and the impact of supplemental *Clj* cells for *Cac* and *Clj* cocultures in bioreactors. The vertical, short-dashed lines represent glucose additions to the bioreactors at 12 and 19 hours. (A-B) The glucose consumption kinetics of the pH 5.5 (A) and pH 5.9 (B) cocultures with ("drop to 5") and without ("stay at") a pH setpoint drop to 5.0 and additional *Clj* (+*Clj*; no additional *Clj* is -*Clj*). Glucose was fed twice at 12 and 19 hours. (C-D) Acetate kinetics of the pH 5.5 (C) and pH 5.9 (D) cocultures. (E-F) Butyrate kinetics at pH 5.5 (E) and pH 5.9 (F). (G-H) Ethanol kinetics at pH 5.5 (G) and pH 5.9 (H).

3 Figure S3

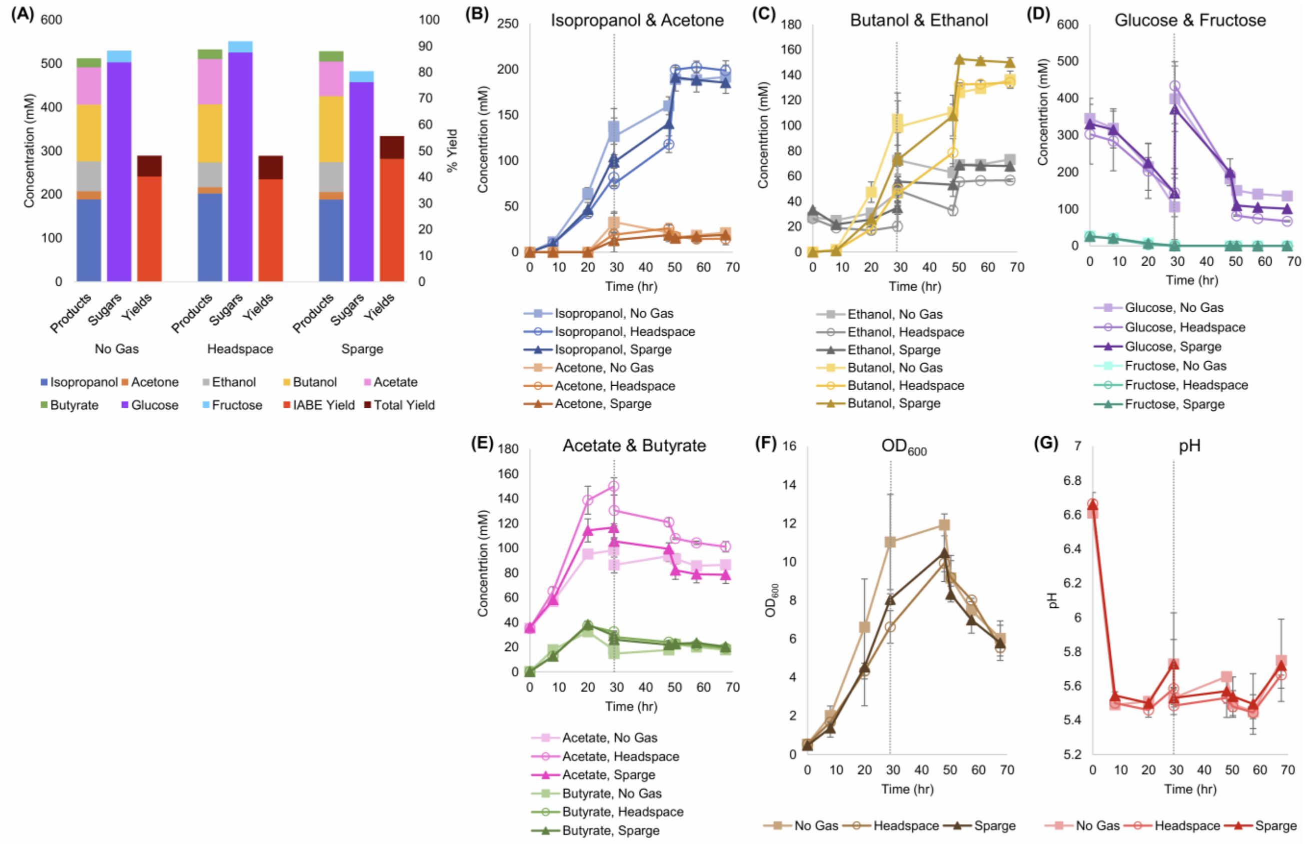

Figure S3: Metabolite production and yields, and sugar consumption, in bioreactor experiments to test gassing configurations for *Cac* and *Clj* cocultures. A blend 85% N_2, 10% CO_2, and 5% H_2 was either sparged (bubbled) into the bioreactors, added to its headspace, or withheld (no gas) except during sampling. The vertical, short-dashed lines represent glucose additions to the bioreactors at 30 hours. (A) Summary of the metabolite concentrations and yields, and sugar consumption. IABE yield refers to IPA, BuOH, EtOH, and acetone. The total yield also includes butyrate and acetate. (B) IPA and acetone concentration kinetics. (C) EtOH and BuOH formation kinetics. (D) Glucose and fructose consumption kinetics. (E) Acetate and butyrate concentration kinetics. (F) Biomass (OD_{600}) kinetic profiles. (G) pH kinetic profiles.

4 Figure S4

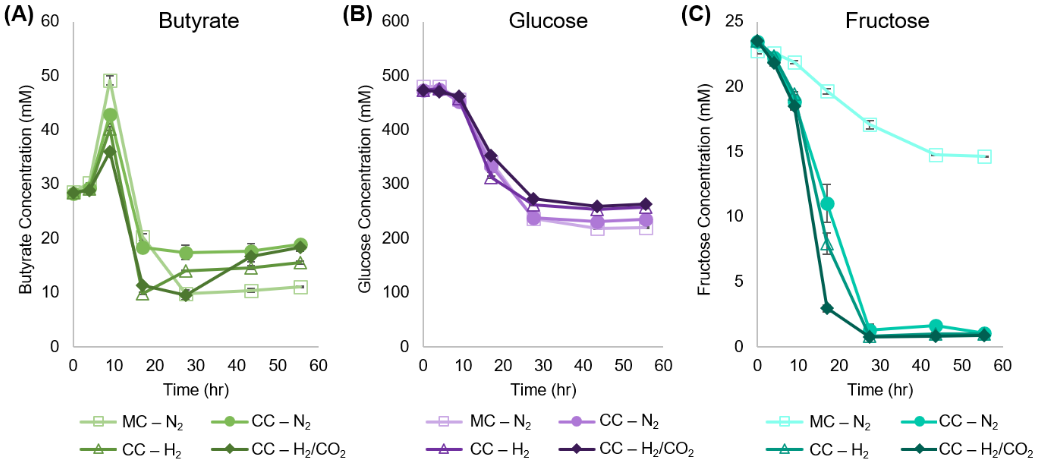

Figure S4: Butyrate and sugar kinetic profiles of monocultures (MC) and cocultures (CC) in sealed serum bottles (the experiments of Figure 2) with different headspace gas composition: N_2, H_2 or Mix [H_2/CO_2 (80/20)]. (A) Butyrate concentration kinetics. (B) Glucose consumption kinetics. (C) Fructose consumption kinetics.

5 Figure S5

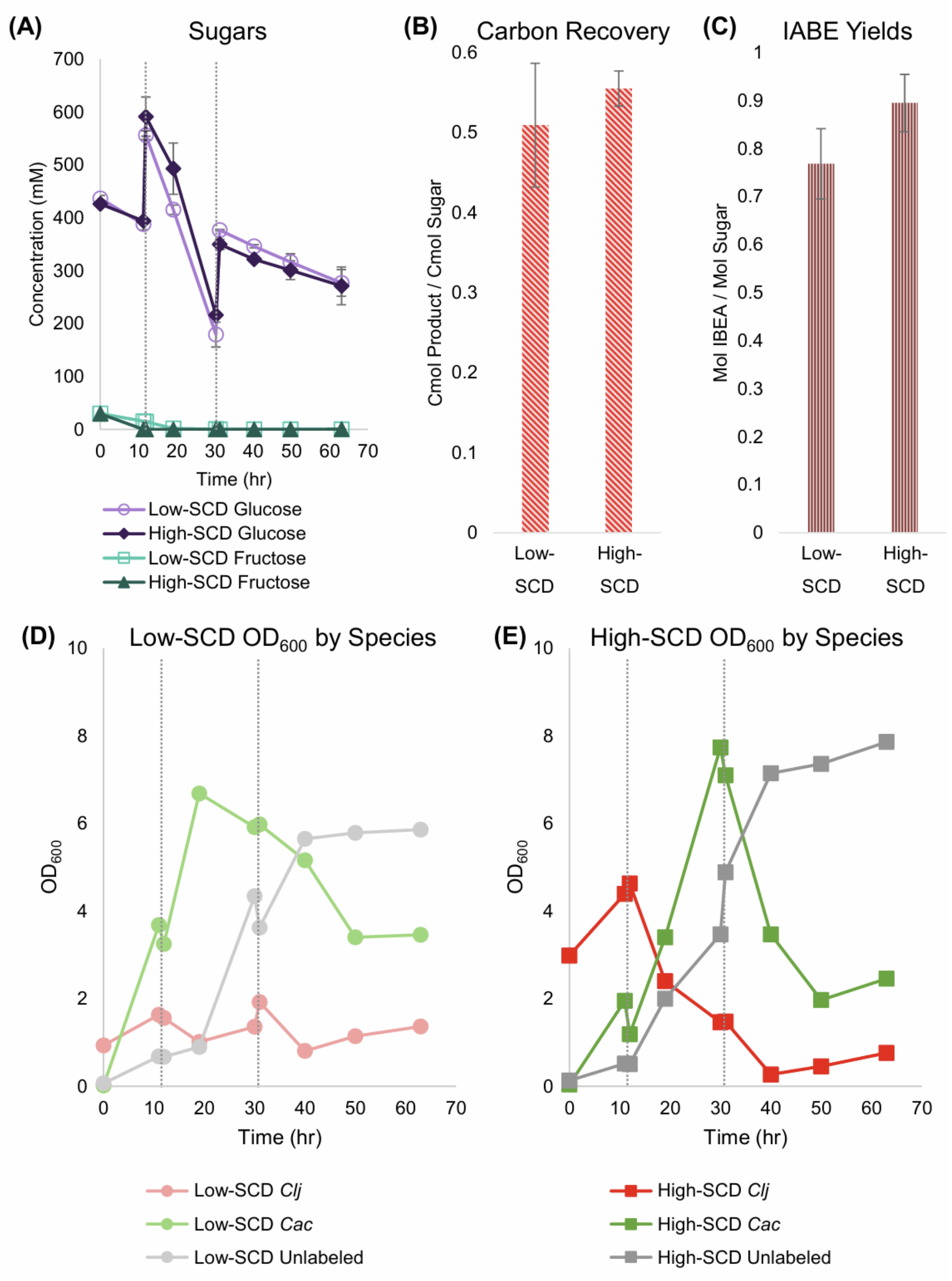

Figure S5: Sugar consumption, carbon recovery and IABE yields, and biomass (OD_{600}) of each species of the bioreactor cocultures of Figure 6 to assess the impact of low or high Starting Cell Densities (SCDs). The vertical, short-dashed lines represent glucose additions, at 10 and 30 hours, to the bioreactors. (A) Sugar consumption kinetics. (B) The carbon-moles (C-mols) of products produced per C-mols of sugars consumed. (C) Mol of alcohols (IABE) produced per mols of sugar consumed. (D-E) The biomass (OD_{600}) kinetics for each coculture species in the low-SCD (D) and high-SCD (E) bioreactors, as determined by the total OD_{600} and the percentage of each species determined by RNA-FISH labeling.
